# Anti-Homer-3 antibodies in cerebrospinal fluid and serum samples from a 58-year-old woman with subacute cerebellar degeneration and diffuse breast adenocarcinoma

**DOI:** 10.1186/s42466-022-00194-9

**Published:** 2022-07-25

**Authors:** Christof Klötzsch, Matthias Böhmert, Ruxandra Hermann, Bianca Teegen, Kristin Rentzsch, Andreas Till

**Affiliations:** 1 Department of Neurology, Hegau-Bodensee-Hospital, Virchowstrasse 10, 78224 Singen, Germany; 2 Clinical Immunological Laboratory Prof. Dr. Med. Winfried Stöcker, Seekamp 31, 23560 Lübeck, Germany; 3 Institute of Experimental Immunology, affiliated to EUROIMMUN Medizinische Labordiagnostika AG, Seekamp 31, 23560 Lübeck, Germany

**Keywords:** Cerebellar degeneration, Paraneoplastic tumour, Homer-3, Anti-Homer-3 antibody, Downbeat nystagmus, Case report

## Abstract

**Introduction:**

Subacute cerebellar ataxia combined with cerebrospinal fluid (CSF) pleocytosis is the result of an immune response that can occur due to viral infections, paraneoplastic diseases or autoimmune-mediated mechanisms. In the following we present the first description of a patient with anti-Homer-3 antibodies in serum and CSF who has been diagnosed with paraneoplastic subacute cerebellar degeneration due to a papillary adenocarcinoma of the breast.

**Case presentation:**

A 58-year-old female was admitted to our clinical department because of increasing gait and visual disturbances starting nine months ago. The neurological examination revealed a downbeat nystagmus, oscillopsia, a severe standing and gait ataxia and a slight dysarthria. Cranial MRI showed no pathological findings. Examination of CSF showed a lymphocytic pleocytosis of 11 cells/µl and an intrathecal IgG synthesis of 26%. Initially, standard serological testing in serum and CSF did not indicate any autoimmune or paraneoplastic aetiology. However, an antigen-specific indirect immunofluorescence test (IIFT) revealed the presence of anti-Homer-3 antibodies (IgG) with a serum titer of 1: 32,000 and a titer of 1: 100 in CSF. Subsequent histological examination of a right axillary lymph node mass showed papillary adenocarcinoma cells. Breast MRI detected multiple bilateral lesions as a diffuse tumour manifestation indicative of adenocarcinoma of the breast. Treatment with high-dose methylprednisolone followed by five plasmaphereses and treatment with 4-aminopyridine resulted in a moderate decrease of the downbeat nystagmus and she was able to move independently with a wheeled walker after 3 weeks. The patient was subsequently treated with chemotherapy (epirubicin, cyclophosphamide) and two series of immunoglobulins (5 × 30 g each). This resulted in a moderate improvement of the cerebellar symptoms with a decrease of ataxia and disappearance of the downbeat nystagmus.

**Conclusion:**

The presented case of anti-Homer-3 antibody-associated cerebellar degeneration is the first that is clearly associated with the detection of a tumour. Interestingly, the Homer-3 protein interaction partner metabotropic glutamate receptor subtype 1A (mGluR1A) is predominantly expressed in Purkinje cells where its function is essential for motor coordination and motor learning. Based on our findings, in subacute cerebellar degeneration, we recommend considering serological testing for anti-Homer-3 antibodies in serum and cerebrospinal fluid together with tumor screening.

## Introduction

Subacute cerebellar ataxia with cerebrospinal fluid (CSF) pleocytosis can be autoimmune-mediated, occur after viral infections or be a symptom of paraneoplastic diseases. The spectrum of onconeural antibodies representing markers for paraneoplastic cerebellar degeneration has been continuously expanded in the past 20 years [1–4]. A small number of relevant autoantibodies have been identified in patients with non-paraneoplastic cerebellar degeneration: glutamic acid decarboxylase [5], metabotropic glutamate receptor type 1 (mGluR1) [6, 7], contactin-associated protein 2 (CASPR2) [8], Rho GTPase activating protein 26/ARHGAP26 [9], inositol-1,4,5-trisphosphate receptor type 1/ITPR1 [10] and, most recently, Homer-3 [11–14]. The Homer-3 protein can be detected almost exclusively perisynaptically in the cerebellum. Strikingly, the intracellular domain of the metabotropic glutamate receptor type 1 (a cell surface receptor strongly expressed perisynaptically on Purkinje cell dendrites) interacts with Homer-3 thus enabling multimerization and clustering of the receptor, a process that is crucial for neuroadaptation [15–18]. So far, a total of only eight cases of idiopathic cerebellitis with subacute cerebellar degeneration associated with the detection of anti-Homer-3 antibodies have been published [11–14], six of which were recently described by in an encephalitis case series in China [14]. One of these patients revealed pulmonary nodules of potential malignancy in chest-CT, but refused biopsy. Because no associated tumour was detectable in any of these patients, a non-paraneoplastic autoimmune-mediated pathogenesis was suspected. All patients showed CSF pleocytosis and an inconsistent response to immunosuppressive therapy. To the best of our knowledge, we present the first case of a patient diagnosed with subacute cerebellar degeneration with anti-Homer-3 antibody in serum and CSF who has histological detection of a papillary adenocarcinoma of the breast.

## Case presentation

**Fig. 1:**
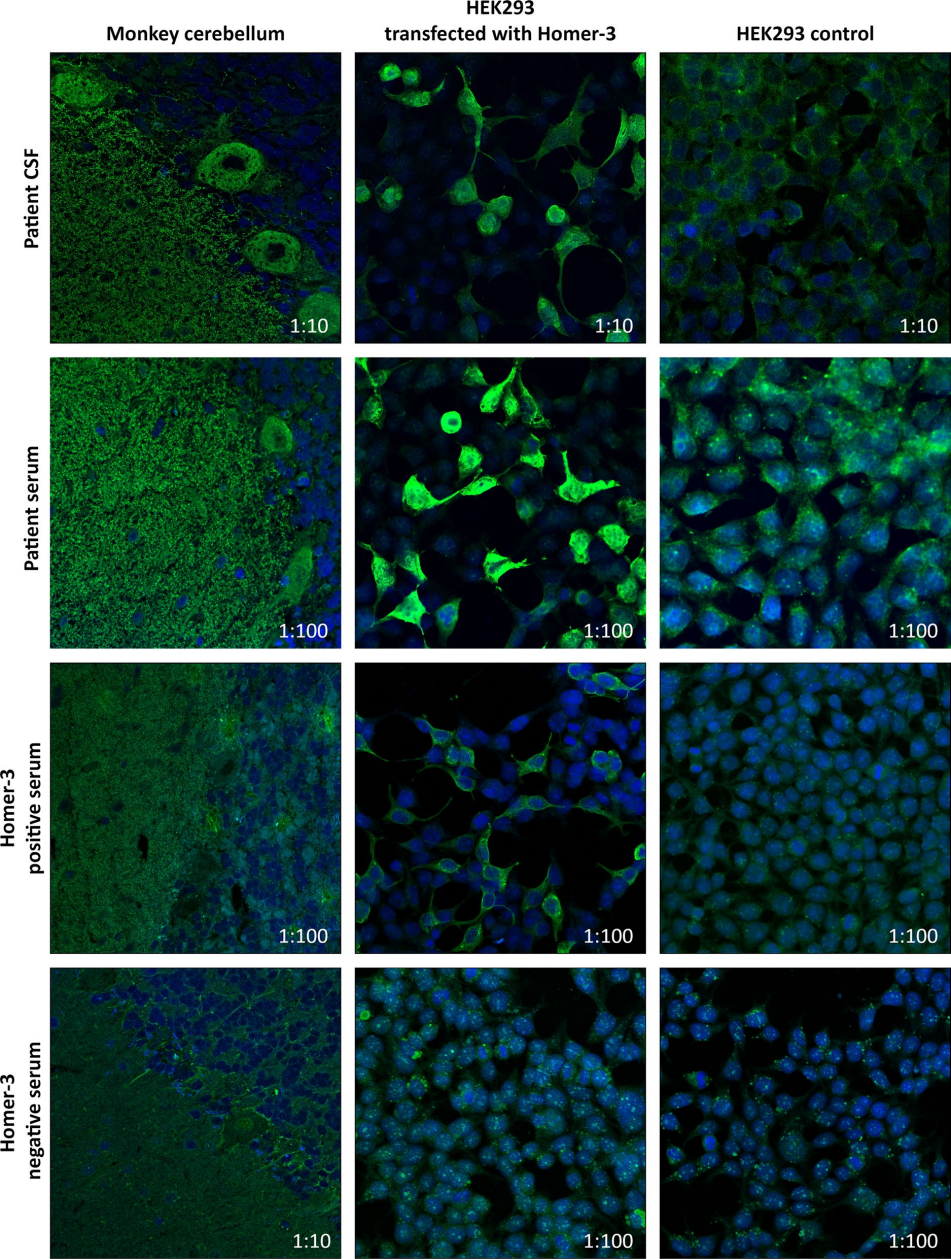
Immunofluorescence analysis of the patient’s samples (rows 1 and 2), as well as two additional samples from two other patients, known to be positive (row 3) or negative (row 4) for anti-Homer-3 antibodies. Numbers in figure panels indicate respective dilution of samples. HEK293 controls were included for all incubated samples (right column) and did not show fluorescence

A 58-year-old female was admitted to the Department of Neurology in February 2019 due to increasing gait and visual disturbances, dizziness and nausea, which she had been suffering from for approximately nine months. Neurological examination revealed downbeat nystagmus with oscillopsia, severe standing and gait ataxia, slight extremity ataxia and slight dysarthria. The patient was only able to walk with assistance. Cranial MRI showed no evidence of cerebellar atrophy. There were no abnormalities in routine laboratory tests. CSF analysis showed pleocytosis of 11 cells/µl and an intrathecal IgG synthesis of 26% (i.e. IgG index = 0.89). Serological tests were negative for autoantibodies against the neuronal antigens GQ1b, aquaporin-4, MOG, GAD, Hu, Yo, Ri, CV2, Ma1, Ma2, NMDA, GABA(A), GABA(B), CASPR2, LGI-1 and mGluR1 receptors. So were serological test for autoantibodies against ITPR1, ARHGAP26 and CARP VIII and Tr/DNER (data not shown). In addition, the following tumour markers were on normal levels: CA 72–4, Cyfra 21–1, NSE, beta-HCG, beta-2-Microglobulin, alpha-Fetoprotein, CA 15–3, CA 19–9, CA 125 and CEA. Indirect immunofluorescence test (IIFT) on HEK293 cells ectopically expressing selected antigens (EUROIMMUN Medizinische Labordiagnostika AG, Lübeck, Germany) revealed the presence of anti-Homer-3 antibodies (IgG) with a titer of 1:32,000 in serum and 1:100 in CSF. IIFT results are shown in Fig. 1, where monkey cerebellum incubated with the serum or CSF of the patient shows a dense, net-like fluorescence of the molecular layer with involvement of the Purkinje cells and accentuation of their neurites. This is also the case for a sample from another Homer-3 positive patient, that was included as control. The sample from an anti-Homer-3 antibody negative patient does not show fluorescence at the 1:10 cut-off on the monkey cerebellum substrate. As already mentioned above, antibodies in the serum and CSF of the patient described in this case report bind the antigen Homer-3 overexpressed in HEK293 cells. This is also true for the additional anti-Homer-3 positive patient sample. Importantly, the HEK293 controls do not show fluorescence, and Homer-3 transfected HEK293 cells do not show fluorescence when incubated with the anti-Homer-3 negative serum.

The thorax and abdomen CT scan revealed a lymph node mass in the right axillary. Histological analysis revealed lymph node metastasis of a papillary adenocarcinoma. Because of unremarkable findings in mammography, a breast-MRI was performed, which showed multiple lesions on both sides as a diffuse tumour manifestation of adenocarcinoma of the breasts. The whole-body tumour PET showed signal increases in both breasts without further tumour manifestations. The patient received four times 1000 mg methylprednisolone followed by five plasmaphereses. To treat the downbeat nystagmus, 4-aminopyridine was dosed with 10 mg b.d. [15], which resulted in a moderate decrease of the nystagmus. After three weeks, the patient’s ambulation improved and she was able to move independently with a wheeled walker. She was subsequently treated with chemotherapy (epirubicin, cyclophosphamide) and two series of immunoglobulins (5 × 30 g each). Until September 2019, there was a moderate improvement of the cerebellar symptoms with a decrease of ataxia, disappearance of the downbeat nystagmus and a decrease in serum anti-Homer-3 antibody titer down to 1:100 in serum.

## Discussion

Since 2003, eight case reports on patients with idiopathic, subacute cerebellar degeneration and anti-Homer-3 antibodies in serum and CSF have been published [11–14]. All patients showed a similar clinical picture with subacute onset ataxia, CSF pleocytosis and/or intrathecal IgG-synthesis and normal findings or cerebellar atrophy on cranial MRI. An extensive search for tumours in these patients did not reveal any underlying malignancy that could have initiated the aberrant production of autoantibodies. Hence, the authors of these studies concluded that anti-Homer-3 antibodies are associated with non-paraneoplastic cerebellar degeneration. To our knowledge, the current case of an anti-Homer-3 antibody-positive woman with severe cerebellar ataxia is the first case that is clearly associated with the detection of a carcinoma. It is important to point out, that we can not entirely exclude the possibility that the presence of anti-Homer-3 IgG in this cancer patient represents an accidental co-occurence without causative connection. While this appears unlikely, future studies may aim to show that the carcinoma cells do in deed express Homer-3 at significant levels and that patient-derived antibodies bind these structures.

The Homer-3 protein displays predominant expression in the dendrites of Purkinje cells. The metabotropic glutamate receptor subtype 1 (mGluR1) is also a predominantly abundant splice variant in Purkinje cells and has a carboxyl-terminal domain that interacts with Homer scaffold proteins [16]. Strikingly, mGluR1 is essential for motor coordination and motor learning [17]. While the potential pathogenic role of Homer-3 in the context of cerebellar ataxias is unknown, we speculate that selective loss of Purkinje cells in the cerebellar cortex due to autoimmune-related mechanisms may contribute to the observed clinical features. Since Homer-3 represents the major scaffold for proteins controlling the intracellular levels of calcium anions (such as glutamate receptor subunits and voltage-dependent calcium channels [18]), future studies may aim at addressing the role of Homer-3 and its protein interaction partners in Purkinje cell—specific calcium homeostasis, especially in the context of cerebellar ataxia. Intriguingly, both anti-Homer-3 and anti-mGluR1 antibodies belong to a group of more than twelve Purkinje cell—specific autoantibodies that are also commonly referred to as ‘Medusa head antibodies’ due to their characteristic dendritic binding pattern in immunohistochemistry [2–4, 19]. Implementing the analysis of these autoantibodies into the diagnostic work-up of patients with cerebellar ataxia of unclear aetiopathogenesis may emerge as a valuable addition to already existing diagnostic routines. The cerebellar symptoms of our patient resemble those of previously published cases with idiopathic anti-Homer-3 antibodies associated with subacute cerebellar degeneration [11–14]. In our view, it is particularly important that they cannot be differentiated clinically from other types of paraneoplastic cerebellar degeneration. All of these cases had an acute to subacute onset with initial light-headedness and/or dizziness and in the further course developed classical cerebellar symptoms such as severe ataxia, dysarthria, downbeat nystagmus and oscillopsia. A detailed analysis of the cerebrospinal fluid frequently showed pleocytosis, increased protein levels and intrathecal IgG synthesis.

Cerebellar atrophy usually occurs in later stages of the disease. It remains unclear whether the only moderate response to immunotherapy is due to Purkinje cell loss during the early stage of the disease and whether stronger immunotherapy in that early phase could have prevented irreversible damage to the Purkinje cells [20].

Testing for the presence of well-characterized autoantibodies against neural antigens is important for the diagnostic work-up in patients with cerebellar ataxia to determine a possible paraneoplastic or autoimmune cause. We highly recommend considering serological testing for the presence of anti-Homer-3 antibodies in serum and CSF accompanied by tumor screening in patients with subacute cerebellar degeneration.

## Data Availability

Single subject data were generated through electronic medical reports and lab results; primary data for MRI und antibody panels are available upon request.

## Abbreviations

b.d.: Twice daily
CASPR2: Contactin-associated protein 2
CEA: Carcinoembryonic antigen
CSF: Cerebrospinal fluid
CT: Computed tomography
GABA: Gamma-aminobutyric acid
GAD: Glutamic acid decarboxylase,
IgG: Immunoglobulin G
IIFT: Indirect immunofluorescence test
LGI-1: Leucine-rich glioma-inactivated protein 1
MOG: Myelin oligodendrocyte glycoprotein
mGluR1: Metabotropic glutamate receptor type 1
MRI: Magnetic resonance imaging
NMDA: N-methyl-D-aspartate
NSE: Neuron-specific enolase
PET: Positron emission tomography

## References

[1] Shams’ili S, Grefkens J, de Leeuw B, van den Bent M, Hooijkaas H, van der Holt B, Vecht C, Sillevis Smitt P (2003). Paraneoplastic cerebellar degeneration associated with antineuronal antibodies: Analysis of 50 patients. Brain.

[2] Jarius S, Wildemann B (2015). 'Medusa head ataxia': The expanding spectrum of Purkinje cell antibodies in autoimmune cerebellar ataxia. Part 1: Anti-mGluR1, anti-Homer-3, anti-Sj/ITPR1 and anti-CARP VIII. Journal od Neuroinflammation.

[3] Jarius S, Wildemann B (2015). 'Medusa head ataxia': The expanding spectrum of Purkinje cell antibodies in autoimmune cerebellar ataxia Part 2: Anti-PKC-gamma, anti-GluR-delta2, anti-Ca/ARHGAP26 and anti-VGCC. Journal of Neuroinflammation.

[4] Jarius S, Wildemann B (2015). 'Medusa head ataxia': the expanding spectrum of Purkinje cell antibodies in autoimmune cerebellar ataxia. Part 3: Anti-Yo/CDR2, anti-Nb/AP3B2, PCA-2, anti-Tr/DNER, other antibodies, diagnostic pitfalls, summary and outlook. Journal of Neuroinflammation.

[5] Saiz A, Blanco Y, Sabater L, González F, Bataller L, Casamitjana R, Ramió-Torrentà L, Graus F (2008). Spectrum of neurological syndromes associated with glutamic acid decarboxylase antibodies: Diagnostic clues for this association. Brain.

[6] Sillevis Smitt P, Kinoshita A, De Leeuw B, Moll W, Coesmans M, Jaarsma D, Henzen-Logmans S, Vecht C, De Zeeuw C, Sekiyama N, Nakanishi S, Shigemoto R (2000). Paraneoplastic cerebellar ataxia due to autoantibodies against a glutamate receptor. New England Journal of Medicine.

[7] Coesmans M, Smitt PA, Linden DJ, Shigemoto R, Hirano T, Yamakawa Y, van Alphen AM, Luo C, van der Geest JN, Kros JM, Gaillard CA, Frens MA, de Zeeuw CI (2003). Mechanisms underlying cerebellar motor deficits due to mGluR1-autoantibodies. Annals of Neurology.

[8] Becker EB, Zuliani L, Pettingill R (2012). Contactin-associated protein-2 antibodies in nonparaneoplastic cerebellar ataxia. Journal of Neurology, Neurosurgery and Psychiatry.

[9] Jarius S, Wandinger KP, Horn S, Heuer H, Wildemann B (2010). A new Purkinje cell antibody (anti-Ca) associated with subacute cerebellar ataxia: immunological characterization. Journal of Neuroinflammation.

[10] Jarius S, Scharf M, Begemann N, Stöcker W, Probst C, Serysheva I, Nagel S, Graus F, Psimaras D, Wildemann B, Komorowski L (2014). Antibodies to the inositol 1,4,5-trisphosphate receptor type 1 (ITPR1) in cerebellar ataxia. Journal of Neuroinflammation.

[11] Zuliani L, Sabater L, Saiz A, Baiges JJ, Giometto B, Graus F (2007). Homer 3 autoimmunity in subacute idiopathic cerebellar ataxia. Neurology.

[12] Höftberger R, Sabater L, Ortega A, Dalmau J, Graus F (2013). Patient With Homer-3 antibodies and cerebellitis. JAMA Neurology.

[13] Xu X, Ren H, Li L, Wang J, Fechner K, Guan H (2019). Anti-Homer-3 antibody associated cerebellar ataxia: A rare case report and literature review. Journal of Neuroimmunology.

[14] Liu M, Ren H, Fan S, Zhang W, Xu Y, Zhao W, Guan H (2021). Encephalitis Collaborative Group. Neurological autoimmunity associated with Homer-3 antibody: A case series from China. Neurology Neuroimmunology Neuroinflammation.

[15] Kalla R, Glasauer S, Büttner U, Brandt T, Strupp M (2007). 4-aminopyridine restores vertical and horizontal neural integrator function in downbeat nystagmus. Brain.

[16] Xiao B, Tu JC, Petralia RS, Yuan JP, Doan A, Breder CD, Ruggiero A, Lanahan AA, Wenthold RJ, Worley PF (1998). Homer regulates the association of group 1 metabotropic glutamate receptors with multivalent complexes of homer-related, synaptic proteins. Neuron.

[17] Ohtani Y, Miyata M, Hashimoto K, Tabata T, Kishimoto Y, Fukaya M, Kase D, Kassai H, Nakao K, Hirata T, Watanabe M, Kano M, Aiba A (2014). The synaptic targeting of mGluR1 by its carboxyl-terminal domain is crucial for cerebellar function. Journal of Neuroscience.

[18] Beqollari D, Kammermeier PJ (2013). The interaction between mGluR1 and the calcium channel Cav(2) (1) preserves coupling in the presence of long Homer proteins. Neuropharmacology.

[19] Shiraishi Y, Mizutani A, Yuasa S, Mikoshiba K, Furuichi T (2004). Differential expression of Homer family proteins in the developing mouse brain. The Journal of Comparative Neurology.

[20] Sivera R, Martín N, Boscá I, Sevilla T, Muelas N, Azorín I, Vílchez JJ, Bolonio M, Donat E, Ribes-Koninckx C, Bataller L (2012). Autoimmunity as a prognostic factor in sporadic adult onset cerebellar ataxia. Journal of Neurology.

